# Microbial oncogenesis within the gastric niche: how the gastric microbiota influences *H. pylori*-induced disease progression

**DOI:** 10.3389/fmicb.2025.1691080

**Published:** 2025-11-19

**Authors:** MaKayla S. Lowe, Richard M. Peek

**Affiliations:** 1 Department of Pathology, Microbiology, and Immunology, Vanderbilt University Medical Center, Nashville, TN, United States; 2 Microbe-Host Interactions Training Program, Vanderbilt University, Nashville, TN, United States; 3 Department of Medicine, Vanderbilt University Medical Center, Nashville, TN, United States

**Keywords:** gastric cancer, *Helicobacter pylori*, Epstein-Barr virus (EBV), microbiome, gastric microbes, CagA, gastric disease

## Abstract

Chronic pathogens incur a significant public health burden, contributing to the development of 1 in 5 cancer cases worldwide. *Helicobacter pylori*, a Gram-negative bacterium that colonizes the gastric mucosa, is the strongest known risk factor for gastric adenocarcinoma, the fifth leading cause of cancer-related mortality. *H. pylori* colonizes almost half of the world's population; however, despite its high prevalence, only approximately 1-3% of infected individuals progress to this malignancy. These data suggest that *H. pylori* colonization alone may be insufficient to fully drive oncogenic progression. Previously considered a sterile environment, the stomach is now recognized to harbor a diverse microbial ecosystem, which plays a crucial role in human health and disease. Emerging research highlights the complex interplay between *H. pylori* and the gastric microbiota, with several commensal bacterial species now identified as modulators of disease progression. Clinical data have defined key variations in gastric microbiota composition between *H. pylori*-infected individuals who progress toward gastric cancer and those who simply develop gastritis alone, further suggesting that the gastric microbiota affects cancer risk in synergy with *H. pylori*. In this review, we will discuss microbial species identified within the stomach of *H. pylori*-infected persons that orchestrate detrimental or protective interactions, which influence the host response and alter cancer risk.

## Gastric cancer as a global health burden

Infectious agents comprise a major global public health concern as they contribute to substantial morbidity and mortality across populations (Global Burden of Disease Antimicrobial Resistance Collaborators, 2022). In 2019 alone, 13.7 million deaths were attributed to infectious agents, with bacterial infections accounting for nearly 8 million of these events (Global Burden of Disease Antimicrobial Resistance Collaborators, 2022). Several pathogens also harbor oncogenic potential, accounting for 1 in 5 cancers worldwide (Vandeven and Nghiem, 2014), underscoring the urgent need to understand and ultimately intervene in microbe-driven carcinogenesis. Gastric cancer, the fifth leading cause of cancer-related mortality worldwide, is closely linked to infection by two highly prevalent oncogenic microorganisms: *Helicobacter pylori* and Epstein-Barr virus (EBV), which infect approximately 43% and 90% of adults worldwide, respectively (Raab-Traub, 2007; Wroblewski et al., 2010; Correa and Piazuelo, 2012; White et al., 2014; Bray et al., 2024; Bu et al., 2024; Chen et al., 2024; Hoover and Higginbotham, 2025). Although widespread, the distribution and impact of these pathogens on disease vary by region (Hooi et al., 2017).

**Figure 1:**
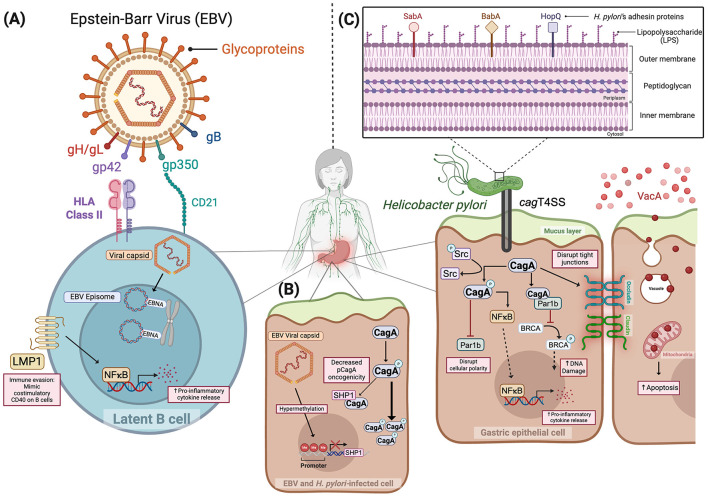
Mechanisms of disease induced by Epstein–Barr virus **(A)**, Epstein-Barr virus and *Helicobacter pylori* **(B)**, and *Helicobacter pylori* **(C)**. Figure created in Biorender.com. gp350, Glycoprotein 350; gp42, Glycoprotein 42; gH/gL, Glycoprotein H, Glycoprotein L; gB, Glycoprotein B; LMP1: Latent membrane protein; EBNA, EBV nuclear antigen; NFκB, Nuclear factor kappa B; Src, Proto-oncogene tyrosine-protein kinase; SHP1, Src homology 2 domain-containing phosphatase 1; SabA, Sialic acid binding Adhesin; BabA, Blood group antigen binding Adhesin; HopQ, *H. pylori* outer membrane Q; CagA, Cytotoxin-associated gene A; *cag*T4SS, Cytotoxin-associated gene type 4 secretion system; VacA, Vacuolating cytotoxin A; Par1b, Polarity-regulating kinase partitioning-defective 1b; BRCA: Breast cancer gene; HLA: Human leukocyte antigen; CD21: Cluster of differentiation 21.

EBV is a ubiquitous DNA virus belonging to the human herpesvirus family and is primarily transmitted via saliva (Raab-Traub, 2007; White et al., 2014; Hoover and Higginbotham, 2025). Following transmission, EBV establishes lifelong latent infection within epithelial cells and B lymphocytes (IARC, 2012; Munz, 2025). Through transcytosis across mucosal epithelial membranes, EBV gains entry to submucosal secondary lymphoid tissue where it directly infects B lymphocytes, utilizing viral glycoproteins to bind cognate host cell receptors (Munz, 2025). Specifically, EBV glycoproteins gp350, gB, and the gp42-gH/gL complex attach to host membrane-bound CD21, neurophilin 1, and MHC class II receptors on B cells, respectively, facilitating viral entry (Escalante et al., 2022; Munz, 2025). To infect epithelial cells, EBV employs similar mechanisms that utilize gp350 and gB to bind to their respective host receptors (Munz, 2025). However, gH/gL engages with Ephrin Receptor A2 (EphA2) independent of gp42. It has also been reported that EBV virions, which infect epithelial cells, express reduced levels of gp42, thereby promoting preferential binding of gH/gL to EphA2 (Munz, 2025). These interactions enable viral-host cell membrane fusion and subsequent viral capsid release. Viral capsids migrate along microtubules to the nucleus where its dsDNA is released and circularizes to form episomes (Munz, 2025). Within the nucleus, EBV expresses latency-associated genes such as Epstein-Barr nuclear antigens (EBNAs) to encode effectors that bind episomes to host chromosomes and latent membrane proteins (LMP) responsible for mimicking B cell costimulatory receptors (Raab-Traub, 2007; Munz, 2025). Concurrently, EBV genetic material is incorporated into host cell DNA to establish and maintain host cell infection (Chesnokova et al., 2009; Huang et al., 2023) (Figure 1A). Based upon the ability to evade host immune surveillance strategies and regulate B cell homeostasis (Cohen, 2003; IARC, 2012; Taylor et al., 2015; Hoover and Higginbotham, 2025), EBV has been implicated in the development of several malignancies, including nasopharyngeal carcinoma, Burkitt lymphoma, Hodgkin's lymphoma, and gastric cancer (Damania et al., 2022). Despite its oncogenic capacity, there are currently no efficacious vaccine strategies or curative antiviral therapies available for the prevention or treatment of EBV infection (Damania et al., 2022). Pharmacological viral inhibitors and B cell-depleting therapies have been employed to manage infection; however, complete viral elimination has not been demonstrated (Munz, 2025). Immune checkpoint blockade of T cell receptors has shown varying clinical responses depending on disease type or location of infection. In Hodgkin's lymphoma, patients experience 80-90% clinical response rates whereas patients with nasopharyngeal cancer exhibit clinical response rates of only 30-40% unless therapies are administered in conjunction with radiation or chemotherapy (Munz, 2025). Ongoing clinical trials are seeking to optimize and develop novel T-cell targeted immunotherapies to mitigate infection (Munz, 2025). However, with such limited treatment options currently available, development of prophylactic immunizations is imperative, specifically for uninfected individuals. Previously developed gp350-targeted vaccines have showed promising effects through decreasing the incidence of mononucleosis, although they failed to completely prevent infection (Cui et al., 2013; Munz, 2025). The EBV glycoproteins, gB and gH/gL, have been identified as attractive targets for vaccine development due to their critical roles in mediating both B lymphocyte and epithelial cell infiltration (Cui et al., 2021; Munz, 2025). Indeed, *in vivo* studies have shown that administration of multimeric forms of these glycoproteins conferred infection resistance to EBV infection in rabbits challenged post-immunization, supporting them as novel targets (Cui et al., 2016; Munz, 2025).

In contradistinction to EBV, *H. pylori* is more prevalent in developing countries, which is likely due to inadequate sanitation and suboptimal hygiene conditions (Damania et al., 2022). The highest rates of *H. pylori* infection occur in Africa, Asia, and South America, with gastric cancer incidence rates typically mirroring infection rates (Hooi et al., 2017; Damania et al., 2022; Thrift et al., 2023). Alarmingly, gastric cancer incidence and mortality rates are also increasing in the United States, specifically among young female and Hispanic male populations (Siegel et al., 2015; Thrift et al., 2023). Universal antimicrobial eradication strategies to prevent gastric cancer are impractical due to the high prevalence of infection, expense, and the side effects of therapy (Polk and Peek, 2010; Anderson et al., 2018). Additionally, carriage of certain *H. pylori* strains is inversely related to the risk of esophageal adenocarcinoma, atopic diseases, and inflammatory bowel disease (Polk and Peek, 2010; Anderson et al., 2018). Since gastric cancer is frequently diagnosed at advanced stages, with a 5 year relative survival rate of 37% at regional stage disease (The American Cancer Society, 2025), these data underscore the critical need for defining the improved mechanistic underpinnings of gastric carcinogenesis.

Interestingly, several studies have revealed a modulatory interspecies relationship between these two well-established oncogenic pathogens. Saju *et al*. reported an agonistic role of EBV on *H. pylori* virulence via altering host protein expression (Saju et al., 2016). Src homology 2 domain-containing phosphatase 1 (SHP1) is a host tyrosine-protein phosphatase, responsible for dephosphorylating the *H. pylori* oncoprotein CagA, and reducing its pathogenic intracellular effects. *In vitro* co-infection with both EBV and *H. pylori* resulted in the downregulation of SHP1 expression via hypermethylation of the SHP1 promoter region by EBV, leading to elevated levels of phosphorylated CagA (Saju et al., 2016) (Figure 1B). Although this model illustrates a prototype of co-infection and virulence, evidence has shown a variety of additional microbial constituents associated with gastric cancer. The following discussion highlights the roles of these injury-augmenting and protective microbial relationships.

## *Helicobacter pylori* infection and virulence

*Helicobacter pylori* is a Gram-negative, microaerophilic bacterium first cultured by Nobel laureates, Barry Marshall and Robin Warren in 1982 (Warren and Marshall, 1983; Marshall and Warren, 1984). Dr. Marshall's self-inoculation provided real-time evidence of the ability of this bacterium to induce gastric inflammation and injury (Marshall et al., 1985), and *H. pylori* was subsequently deemed a class I carcinogen by the World Health Organization's International Agency for Research on Cancer (IARC, 1994). Neutralophilic by nature, *H. pylori* harbors urease, which catalyzes the hydrolysis of urea into ammonia (NH_3_) and carbon dioxide (CO_2_), which serves to neutralize gastric acidity and permit chronic colonization of the harsh gastric environment (Sirit and Peek, 2025). Coupled with the bacterium's helical morphology and flagellated motility to aid with penetration of the gastric mucus layer and enable direct interaction with gastric epithelial cells, *H. pylori* achieves successful colonization and establishes persistent infection unless eradicated effectively through directed antibiotic treatment (Sirit and Peek, 2025).

The pathogenic potential of *H. pylori* is highly strain dependent, and isolates that possess the cytotoxin-associated gene (*cag*) pathogenicity island (*cag* PAI), which encodes a type IV secretion system (T4SS), are more highly associated with gastric cancer (Wroblewski et al., 2010; Cover et al., 2020). This complex apparatus translocates bacterial effectors into host epithelial cells to assist in nutrient acquisition and colonization by disrupting several epithelial cellular pathways (Cover et al., 2020) (Figure 1C). Among these translocated effectors, CagA is the most well-characterized and is classified as a microbial oncoprotein (Backert et al., 2015). Upon entering host cells, CagA is phosphorylated by Src family kinases at EPIYA motifs, which triggers downstream signaling pathways that disrupt cellular polarity, promote proliferation, and enhance cell migration (Amieva et al., 2003; Saadat et al., 2007; Backert et al., 2015). Specifically, phosphorylated-CagA binds to and interacts with host effectors, including SHP1 protein tyrosine phosphatases and Par1b kinases (Mishra et al., 2015; Imai et al., 2021). Through inhibiting the phosphorylating capacity of Par1b, CagA hinders the activation and subsequent translocation of the breast cancer gene, BRCA, into the nucleus to repair damaged DNA. This negative regulation also impedes the ability of Par1b to regulate cellular polarity and epithelial-mesenchymal transition (Imai et al., 2021). Moreover, our group and others have demonstrated that non-phosphorylated CagA exerts pathologic effects, including ß-catenin activation (Amieva et al., 2003; Franco et al., 2005; Wroblewski et al., 2019). Beyond its disruptive direct molecular interactions, CagA promotes chronic inflammation, a key driver of tumorigenesis, through activation of NF-κB and induction of IL-8 chemokine signaling (Fazeli et al., 2016; Duan et al., 2025). Additional substrates translocated by the *cag* PAI, such as DNA, ADP-heptose, peptidoglycan, and lipopolysaccharide (LPS), are recognized by host pattern recognition receptors; however, the cognate downstream signaling effects induced by these effectors in the gastric epithelium remain less clear (Varga et al., 2016; Pfannkuch et al., 2019).

The pathogenic potential of *H. pylori* also extends beyond cag T4SS activity. For example, a diverse range of outer membrane proteins serve as adhesins, including CagL, HopQ, SabA, and BabA, which are critical for the attachment of *H. pylori* to the gastric epithelium (Backert et al., 2011; Sirit and Peek, 2025) (Figure 1C). Secretion of vacuolating cytotoxin A (VacA), a pore-forming protein that induces vacuoles within epithelial cells following endocytosis, disrupts mitochondrial function, cellular signaling, and epithelial integrity (Cover and Blanke, 2005). Importantly, VacA also exerts deleterious effects on host immune cells, which include disruption of phagosome maturation in macrophages and antigen presentation by B lymphocytes and attenuation of T cell activation and effector T cell expansion, all of which facilitate immune evasion by *H. pylori* (Cover and Blanke, 2005; Djekic and Muller, 2016).

In a subset of patients, chronic *H. pylori* infection initiates a well-characterized histopathological progression known as the Correa cascade (Correa and Piazuelo, 2012). Dr. Pelayo Correa initially described this pathway as progressing from non-atrophic gastritis to chronic atrophic gastritis, intestinal metaplasia, dysplasia, and eventually gastric adenocarcinoma (Correa and Piazuelo, 2012). However, although *H. pylori* is a well-established driver of this carcinogenic cascade, only 1-3% of infected individuals ultimately progress to gastric cancer (Correa and Piazuelo, 2012). This striking disparity highlights the importance of defining additional constituents that play critical roles in modulating oncogenesis, which include host genetic variation, immune responses, dietary exposures, and other exposomal influences (Wroblewski et al., 2010; Mendes-Rocha et al., 2023).

Clinical data strongly indicate that the gastric microbiota affects cancer risk in synergy with *H. pylori* (Abreu and Peek, 2014; Mannion et al., 2023). Gastric microbial dysbiosis is significantly more frequent in patients with gastric cancer vs. patients with gastritis alone (Coker et al., 2018; Ferreira et al., 2018; Kwon et al., 2022). Specific differences in the gastric microbiota among persons residing in low-risk versus high-risk regions for gastric cancer have been identified (Yang et al., 2016; Shen et al., 2022). Transplantation of the gastric microbiota from patients with premalignant and malignant lesions into germ-free mice induces gastric dysplasia (Kwon et al., 2022). Finally, antibiotic therapy targeting *H. pylori* significantly reduced the incidence of gastric cancer in high-risk populations, despite failed *H. pylori* clearance in half of treated individuals (Ma et al., 2012). There are several potential mechanisms that may underpin this disparity. For example, alteration of the gastric microbiota by antibiotics could attenuate carcinogenic interactions between members of the gastric microbiome and persistent *H. pylori* strains. Antibiotic treatment could also directly alter *H. pylori* virulence phenotypes of persisting strains in lieu of completely eliminating the bacterium. Antibiotic-induced changes to either the gastric or the intestinal microbiota could directly attenuate cancer development *per se*, irregardless of the presence of *H. pylori*. Finally, antibiotic-induced microbial alterations may influence how dietary components of the exposome interact with the host, contributing to protection against cancer development. However, gastric microbial community structure within the context of gastric carcinogenesis has only been studied in cross-sectional trials, which cannot differentiate cause from effect. As most *H. pylori-*infected persons do not develop cancer, these data suggest that interactions between *H. pylori* and other microbial species may influence oncogenesis (Polk and Peek, 2010; Shen et al., 2022).

In this review, we discuss the gastric microbiota as a modulator of disease progression and elucidate how the interplay of specific gastric microbiota components with *H. pylori* drives or attenuates oncogenesis. We also address existing knowledge gaps concerning non-*H. pylori-*driven mechanisms that underpin gastric oncogenesis and similar malignancies, with a particular emphasis on microbial taxa beyond EBV.

## The composition and role of the gastric microbiota in disease

At birth, maternal microbial species rapidly colonize the gastrointestinal niche, playing a fundamental role in priming physiological responses to the exposome throughout an individual's lifetime, and the gut microbiota continuously evolves until reaching a stable composition around 3 years of age (Rinninella et al., 2019). The gastrointestinal microbiota plays a vital role in maintaining homeostasis through symbiotic interactions with epithelial cells, the lymphoid system, and surrounding microorganisms (Dieterich et al., 2018). Through leveraging nutrient acquisition and bacteriocin secretion, commensal bacteria confer colonization resistance to pathobionts and invasive species (Rinninella et al., 2019). Moreover, the development of immunotolerance to innocuous commensal antigens requires a diverse functional microbial ecosystem as determined by germ-free animal model systems (Maciel-Fiuza et al., 2023; Li Z. et al., 2024). Thus, disruption of this delicately balanced microbial ecosystem and microenvironment facilitates the development of disease (Round and Mazmanian, 2009; Maciel-Fiuza et al., 2023).

Despite the continuity of the gastrointestinal tract, microbial composition and abundances differ across regions and among individuals (Rinninella et al., 2019). Each region of the gastrointestinal tract harbors distinct niche-specific conditions that are defined by oxygen levels, pH, and peristalsis, all of which influence habitability for different microbial communities. *Bacteroidetes* and *Firmicutes* are the dominant phyla present in the large intestine, which is defined by basic pH, low oxygen, and infrequent peristalsis (Rinninella et al., 2019; Maciel-Fiuza et al., 2023). *Actinobacteria* and *Proteobacteria* are also components of the commensal gut microbiota, although at lower abundances. Previously considered a sterile environment, the stomach is now recognized to harbor a diverse microbial ecosystem with a microbial load of approximately 10^1^ colony-forming units per gram (American Society for Microbiology, 2013; Dieterich et al., 2018). In the stomach, low pH and high oxygen concentrations foster conditions that promote a high relative abundance of *Firmicutes* and *Proteobacteria* (Ohno and Satoh-Takayama, 2020).

## Current techniques to investigate the microbiome

Investigations of the microbiome have rapidly advanced through the development of a range of investigative methods and technologies encompassing the profiling of microbial DNA, RNA, protein, and metabolites (Knight et al., 2018; Gotschlich et al., 2019). To reveal the composition of microbial communities, 16S and 18S rRNA amplicon sequencing enables the identification of prokaryotic and eukaryotic taxa, respectively, of specific species based on conserved ribosomal genetic sequences (Johnson et al., 2019). While these techniques provide insight at a genus level, they have limited resolution for differentiating closely related species that share ribosomal sequences (Johnson et al., 2019). Whole metagenomic sequencing (WMS) allows for a more comprehensive characterization of microbial communities through analyzing the entire genome of all microorganisms within a single sample (Gotschlich et al., 2019). This untargeted approach allows for taxonomic resolution at a species- and strain-level, providing detailed information of community composition (Knight et al., 2018). Complementary to this, metatranscriptomic, metaproteomic, and metametabolomic analyses more closely measure the functional activity of the microbiota by quantifying RNA, protein, and metabolite production, which can only be inferred by WMS (Knight et al., 2018).

To complement techniques that develop a detailed itinerary of microbial species, *in vivo* models are critical for investigating the functional biological processes and phenotypes mediated by the microbiota. Gnotobiotic, or germ-free, animal models are devoid of all micro-organisms enabling a direct investigation of specific microorganisms in health and disease (Fiebiger et al., 2016; Luczynski et al., 2016). However, while these models offer great resolution, they are limited by underdeveloped immune and organ systems and the absence of complex microbial communities in such models can be missed (Fiebiger et al., 2016; Luczynski et al., 2016). To overcome this, microbial species-defined resources such as Altered Schaelder Flora (ASF), which contain a defined consortium of bacterial species, offer a simplified microbial gut microbiota to overcome the absence of complex interactions in mono-colonized germ-free models (Wymore Brand et al., 2015). Stem-cell derived organoids offer an *ex vivo* approach to more carefully control microbial interactions in a primary cell model system that more faithfully recapitulates the *in vivo* gastric niche (Pearce et al., 2018). Ultimately, integrating orthogonal multi-omics approaches with *in vivo and ex vivo* methodologies will provide a more comprehensive understanding of defined microbial communities, their activity, and ultimately, their impact on human health.

## Human studies define perturbation of the gastric niche following *H. pylori* infection

Advances in whole-genome sequencing have strengthened the ability to accurately define the microbiota of *H. pylori-*infected individuals, revealing temporal changes linked to disease progression (Weinstock, 2012). External stressors, such as *H. pylori*, disrupt the gastric microbial ecosystem, highlighting the dynamic plasticity of the stomach under various conditions. Following infection, *H. pylori* dominates the gastric niche and reduces the overall microbial diversity. Reduced diversity has been linked to worsened disease outcomes, including the progression toward gastric cancer (Mendes-Rocha et al., 2023). However, the spike in *H. pylori* abundance is not maintained throughout a lifetime (Fakharian et al., 2022), as *H. pylori*-induced hypochlorhydric atrophic gastritis is a key step in the progression to intestinal-type gastric cancer (Correa, 1992), which facilitates colonization of other bacteria. Furthermore, the gastric microbiota has been compared in persons with non-atrophic gastritis versus hypochlorhydric gastric cancer; the microbiota in cancer samples exhibited less diversity and an overabundance of *Citrobacter, Clostridium, Lactobacillus, Achromobacter*, and *Rhodococcus*, species typically found in the intestinal microbiota (Ferreira et al., 2018). In later-stage disease, *H. pylori* abundance declines, which is accompanied by an increase in lactic acid-producing bacteria as well as intestinal and oral commensals (Castano-Rodriguez et al., 2017). Genera such as *Streptococcus, Lactobacillus, and Fusobacterium* species expand concurrently during carcinogenesis, yielding a dysbiotic gastric environment marked by increased species richness and phylogenetic diversity (Shen et al., 2022; Lehr et al., 2023).

The impact of *H. pylori* eradication therapy has also emphasized a potential role of the gastric microbiota in pathogenesis. In a 15 year intervention study, antibiotic treatment targeting *H. pylori* significantly reduced the incidence of gastric cancer, despite the fact that fewer than half of treated individuals remained free of *H. pylori* infection (Ma et al., 2012). These findings suggest that antibiotics that modify the microbiota can attenuate the development of gastric cancer despite the presence of *H. pylori*. Although hypothesis-generating, these data cannot differentiate cause from effect. However, the concept that microbial factors can directly drive a specific phenotype has now been substantiated *in vivo* through the work of Kwon et al. (2022), which demonstrated that transplantation of the gastric microbiota harvested from patients with premalignant and malignant lesions into germ-free mice induces intestinal metaplasia and dysplasia. Taken together, these data indicate that *H. pylori* initiates a decades-long evolution of the human gastric microbiota. Further, technological advances have now enabled the identification of several microorganisms as potential novel oncogenic contributors to or suppressors of *H. pylori*-driven gastric disease progression.

## Beyond *H. pylori:* using animal models to define the influence of non-*H. Pylori* species on gastric carcinogenesis

The complex interplay between microorganisms in the stomach has been shown to exert both protective and pathogenic effects on *H. pylori*-induced gastric disease progression and virulence. Similar to the human stomach, the most abundant microbial phyla in the mouse stomach are *Bacteroidetes, Firmicutes, Proteobacteria*, and *Actinobacteria* and, similar to humans, *H. pylori* induces atrophic gastritis in mice (Lofgren et al., 2011; Ge et al., 2018). The extent of injury induced by *H. pylori* varies depending on the composition of the murine gastric microbiota, with different ratios of *Lactobacillus* species ASF360 and ASF361, as well as different mouse vendors altering the severity of host responses (Rolig et al., 2013; Ge et al., 2018). Gnotobiotic mice provide a compelling model to study *H. pylori* and the microbiota via the ability to incrementally add individual or pooled collections of microorganisms. INS-GAS mice are transgenic, hypergastrinemic mice predisposed to gastric cancer (Correa et al., 1976), and it has been demonstrated that germ-free INS-GAS mice infected with *H. pylori* develop an attenuated progression to neoplasia compared to *H. pylori-*infected INS-GAS mice with a complex microbiota (Lofgren et al., 2011). Thus, rodent models have provided key insights into the role of the gastric microbiota in modifying *H. pylori*-induced disease.

## Pathogenic species: bacterial species that promote *H. pylori* virulence

Identification of bacterial species that are overabundant in gastric cancer is a rapidly evolving field, and geographically-related but epidemiologically-distinct communities offer a valuable clinical framework to investigate how microbial factors shape gastric cancer risk among *H. pylori-*infected individuals. Dr. Pelayo Correa originally observed that the prevalence of *H. pylori* infection is very high throughout Colombia; however, individuals living in the mountains exhibited markedly increased rates of gastric cancer (150 cases/100,000), vs. those on the coast (6 cases/100,000) (Correa et al., 1976; Cuello et al., 1976). This 25-fold disparity in prevalence of gastric cancer, but not *H. pylori*, provided a unique opportunity to identify additional oncogenic constituents, such as population-specific components of the gastric microbiota. In adults residing in either a low-risk (Tumaco) or a high-risk (Túquerres) gastric cancer region in Colombia, significant differences within the gastric microbiota were identified (Shen et al., 2022). Specifically, an oral commensal, *Streptococcus salivarius*, was enriched in *H. pylori-*infected individuals within the high-risk gastric cancer region of Túquerres, Colombia (Shen et al., 2022) (Table 1). *In vivo* co-colonization of *S. salivarius* and *H. pylori in* germ-free INS-GAS mice successfully elicited increased injury and dysplasia as well as elevated pro-inflammatory cytokine expression compared to *H. pylori* mono-colonized mice (Shen et al., 2022) (Table 1).

**Table 1:** Bacterial species that modulate *Helicobacter pylori* virulence and disease progression.

**Microbial role**	**Species**	**Typical niche**	**Mechanism to alter *H. pylori-induced* gastric disease**	**References**
Pathogenic	*Streptococcus salivarius*	Oral	• Increases pro-inflammatory cytokine expression *in vivo* • Exacerbates *H. pylori-*induced injury *in vivo*	Shen et al., 2022
	*Fusobacterium nucleatum*	Oral	• Inhibits apoptosis and increases proliferation of T cells • Induces DNA damage via genotoxins *in vitro* and *in vivo*	Niikura et al., 2023 and Zhang et al., 2025
	*Neisseria subflava*	Upper respiratory tract	• Increases pro-inflammatory response *in vivo*	Niikura et al., 2023
	*Porphyromonas gingivalis*	Oral	• Disrupts epithelial cell barrier and increases pro-inflammatory immune response • Activates anti-apoptotic signaling pathways *in vitro* and *ex vivo*	Bakhti and Latifi-Navid, 2021, Oriuchi et al., 2024, and Xia et al., 2025
	*Streptococcus anginosus*	Gastrointestinal tract	• Induces gastric carcinogenesis through the disruption of MAPK signaling pathway • Induces DNA damage via acetaldehyde secretion	Fu et al., 2024
Protective	*Lactobacillus gasseri*	Oral	• Suppresses *H. pylori* adhesin expression, CagA-induced morphological alterations, and pro-inflammatory cytokine production	de Klerk et al., 2016 and Gupta et al., 2025
	*Lactobacillus brevis*	Oral	• Suppresses *H. pylori* adhesin expression	de Klerk et al., 2016
	*Lactobacillus salivarius*	Oral	• Decreases *H. pylori* colonization *in vivo*	Li J. et al., 2024
	*Lactobacillus reuteri*	Oral	• Increased *H. pylori* eradication rates in patients treated with triple therapy	Ismail et al., 2023
	*Streptococcus mitis*	Oral	• Drives *H. pylori* coccoid morphology *in vitro*	Khosravi et al., 2014
	*Cutibacterium acnes*	Skin	• Decreases *H. pylori-*driven inflammation and cellular proliferation *in vivo*	Lunger et al., 2024
	*Staphylococcus epidermidis*	Skin	• Decreases pro-inflammatory cytokine expression *in vivo*	Shen et al., 2022

Additional bacterial species have also been identified as contributors to the initiation and progression of gastric cancer, independent of *H. pylori*. Microbiome analyses of gastric tissue harvested from a subset of patients post-*H. pylori* eradication revealed a persistent dysbiosis marked by enrichment of *Fusobacterium nucleatum*, an oral commensal species known to be abundant within colorectal cancer tissue, and *Neisseria subflava*, a respiratory tract commensal (Bakhti and Latifi-Navid, 2021; Niikura et al., 2023; Zepeda-Rivera et al., 2024) (Table 1). In these patients, increased gastric cancer incidence was observed, which corresponded to elevated levels of DNA damage and induction of pro-inflammatory responses by these species (Niikura et al., 2023; Zhang et al., 2025). Evidence now suggests that *F. nucleatum* colonizes the intratumoral environment of gastric cancer specimens and recruits tumor-associated neutrophils, which likely facilitate disease progression (Zhang et al., 2025). *Porphyromonas gingivalis*, an oral pathogen associated with periodontal disease, also exerts pathogenic effects when colonizing the gastric niche by disruption of the gastric epithelial barrier, induction of a heightened immune response through increased TNFα release, and activation of macrophages (Xia et al., 2025) (Table 1). Moreover, *P. gingivalis* facilitates oncogenesis via activating anti-apoptotic signaling pathways and inducing cellular proliferation (Bakhti and Latifi-Navid, 2021; Oriuchi et al., 2024).

A novel initiator of gastric disease, *Streptococcus anginosus*, has recently been shown to be overabundant in human gastric cancer specimens compared to non-cancer specimens (Fu et al., 2024) (Table 1). Mechanistically, *S. anginosus* activates mitogen-activated protein kinase (MAPK) signaling via the interaction between surface-bound *Treponema pallidum* membrane protein C (TMPC) and the gastric epithelial Annexin A2 (ANXA2) protein receptor to promote proliferation and inhibit apoptosis (Fu et al., 2024). *S. anginosus* also produces acetaldehyde with the capacity to induce DNA damage and promote immune cell recruitment (Xia et al., 2025). Intratumoral *S. anginosus* increases cellular proliferation, decreases tumor immune microenvironment CD8^+^ T-cell infiltration, and upregulates intratumoral arginine metabolism to produce ornithine and further promote tumor cell growth (Yuan et al., 2024).

Collectively, these findings describe alternative microbial drivers of gastric oncogenesis. However, as described in detail below, several commensal species have been identified that not only exert beneficial responses, but can also drive pathologic responses, depending on host and microbiological context. Thus, defining the precise functional interactions between *H. pylori* and these commensal bacteria remains an active area of investigation, which will be crucial to uncovering risk factors and therapeutic targets for gastric disease intervention.

## Gastric microbial species can also dampen *H. pylori* pathogenicity

*Lactobacillus*, a bacterial genus widely recognized for probiotic effects (Xia et al., 2025), can attenuate *H. pylori* virulence, thereby impacting its oncogenic potential. Identified as human commensals, *L. gasseri* and *L. brevis* exhibit suppressive effects on *H. pylori* adhesion to gastric epithelial cells via downregulating the adhesin SabA (de Klerk et al., 2016) (Table 1). More recently, *L. gasseri* was found to impair the intracellular phosphorylation of CagA and subsequently cellular morphologic alterations (Gupta et al., 2025), as well as suppress pro-inflammatory cytokine secretion by macrophages (Gebremariam et al., 2019). *Ligilactobacillus salivarius*, also known as *Lactobacillus salivarius*, attenuated colonization of *H. pylori* and induction of gastritis in mice (Li J. et al., 2024) (Table 1). Other *Lactobacillus* strains have been shown to inhibit *H. pylori* motility and urease activity (Dempsey and Corr, 2022). In clinical trials, *L*. *reuteri* has been evaluated as an adjunct therapy for standard-of-care triple antibacterial therapy, which improved *H. pylori* eradication rates relative to standard therapy alone (Ismail et al., 2023) (Table 1). While a plethora of *Lactobacillus* strains exert beneficial effects, their production of lactic acid can exert detrimental consequences, specifically within the context of cancer, as metabolism of lactic acid readily generates lactate, which can serve as an energy source for tumors (Castano-Rodriguez et al., 2017; Xia et al., 2025). Physicians often prescribe probiotics containing *Lactobacillus* for therapeutic and preventative care (Kochan et al., 2011). However, although considered a weak pathogen, the ability of *Lactobacillus* to bind host extracellular matrix proteins facilitates interactions with host cells, particularly in immunocompromised individuals (Pararajasingam and Uwagwu, 2017; Rossi et al., 2019). Clinical evidence further highlights the pathogenic potential of probiotic-derived *L. paracasei* and *L. rhamnosus* strains in causing liver abscesses in diabetic patient and sepsis in post-operative patients, respectively (Kochan et al., 2011; Pararajasingam and Uwagwu, 2017). These cases underscore that the opportunistic pathogenic capabilities of *Lactobacillus* may be underestimated and suggest that the duality of this genus to either worsen or attenuate disease should be considered prior to prescribing these agents to immunocompromised populations.

In addition to *Lactobacillus*, other recently recognized bacterial species have been associated with attenuation of *H. pylori* pathogenicity. *Cutibacterium acnes*, also known as *Propionibacterium acnes*, is an antimicrobial thiopeptide-secreting skin commensal which was identified as being over-abundant in a low-gastric cancer risk *H. pylori*-infected Nicaraguan population (Mayslich et al., 2021; Lunger et al., 2024) (Table 1). Further, this species has now been shown to reduce pro-inflammatory and cellular proliferative markers in germ-free INS-GAS mice following co-infection with *H. pylori* compared to *H. pylori* mono-colonized mice. However, like other commensal species, *C. acnes* can also exert detrimental effects under certain conditions (Mayslich et al., 2021). This species is strongly associated with acne vulgaris, where it enhances inflammation through inducing pro-inflammatory signaling via NFκB and MAPK signaling (Mayslich et al., 2021). More recently, it has been recognized as an inducer of Type-1 interferon signaling via the cGAS-STING pathway in human macrophages (Fischer et al., 2020). *Streptococcus mitis* has been previously described to drive *H. pylori* to adopt a dormant coccoid morphology *in vitro* (Khosravi et al., 2014) (Table 1). *Staphylococcus epidermidis*, a skin commensal, was shown to be differentially abundant in the low-gastric cancer risk region of Tumaco, Colombia (Shen et al., 2022; Mannion et al., 2023) (Table 1). Concordantly, co-infection of *S. epidermidis* with *H. pylori* in germ-free INS-GAS mice resulted in reduced pro-inflammatory cytokine expression relative to *H. pylori* mono-colonized mice (Shen et al., 2022). Thus, identification of protective species that suppress *H. pylori* virulence and associated disease provides novel avenues of probiotic-based therapeutic approaches as well as potential prognostic microbial biomarkers.

## Discussion and future perspectives

Microbial oncogenesis remains a persistent challenge in human health (Vandeven and Nghiem, 2014). While fundamental to normal physiology, microorganisms have the potential to promote adverse outcomes under conducive conditions. The role of the gastric microbiota in cancer progression is an emerging area of research. Historically, *H. pylori* was regarded as the singular bacterial driver of disease initiation within the stomach (Chen et al., 2024). However, advances in high-throughput technologies have enabled more refined analyses of gastrointestinal microbial communities, revealing potential favorable and harmful bacterial modulators of disease pathogenesis (Shen et al., 2022; Mannion et al., 2023). Throughout the progression of gastric carcinogenesis, the stomach microbiome undergoes continuous evolution, marked by a decline in diversity over time. Early-stage disease is strongly characterized by a dominant abundance of *H. pylori*, though its gradual reduction, in concurrence with loss of acid-secreting capacity of the stomach over years, enables the expansion of opportunistic pathogens (Plottel and Blaser, 2011). Correlation studies have highlighted distinct patterns of co-occurring and co-excluding gastric bacteria at different stages of gastric cancer development. Human data further support the premise that these shifts may contribute to cancer risk, as such relationships are significantly stronger in patients with gastric cancer compared to patients with gastritis alone (Ferreira et al., 2018; Shen et al., 2022). Collectively, these observations underscore a compelling shift in concept from *H. pylori* functioning as the sole driver of oncogenesis to a working model that incorporates the complex interplay between *H. pylori* and the host microbiota in gastric carcinogenesis. While several species have been recently implicated in disease progression (de Klerk et al., 2016; Gebremariam et al., 2019; Bakhti and Latifi-Navid, 2021; Dempsey and Corr, 2022; Shen et al., 2022; Ismail et al., 2023; Niikura et al., 2023; Fu et al., 2024; Li J. et al., 2024; Lunger et al., 2024; Oriuchi et al., 2024; Yuan et al., 2024; Gupta et al., 2025; Xia et al., 2025; Zhang et al., 2025), a critical knowledge gap remains in the understanding of the precise intra-species mechanisms driving observed phenotypic changes. Leveraging gnotobiotic mouse models, comprehensive multi-omics techniques, and bacterial genetic manipulation to investigate microbe-microbe and host-microbe molecular interactions will ultimately provide a framework for shaping the development of clinical interventions and noninvasive strategies for prognosis. The current standard of care for earlier stages of gastric carcinogenesis begins with *H. pylori* elimination through multi-drug therapy, followed by ongoing surveillance if premalignant lesions are present, coupled with lifestyle changes (Goderska et al., 2018). In contrast, late-stage gastric cancer requires more invasive approaches including surgical resection, radiation, and chemotherapy (Menon et al., 2025). As gastric carcinogenesis is increasingly understood to be influenced by an ever-expanding network of host and microbial factors, critical steps toward more effective therapeutic strategies will likely follow. For example, administration of probiotics containing identified beneficial species, such as *S. epidermidis* or *C. acnes*, as microbial adjuvants to standard of care treatments may enhance disease prevention and/or contribute to disease regression. The impact of microbial monotherapy could also be augmented via delivery of a consortia of beneficial species, such as *L. gasseri* and *S. epidermidis*. Microbiota profiling could also facilitate the development of microbial biomarkers (e.g., high *S. salivarius/S. epidermidis* ratio*)* for gastric cancer risk stratification among *H. pylori-*infected persons. Finally, leveraging and manipulating microbial metabolites such as lactate that drive disease exacerbation, offers another exciting opportunity to mitigate disease. As research continues to identify changes across a myriad of microbial communities, the portfolio of microbes used for assessing risk and offering new therapeutic options will become more precise, allowing for the refinement of tailored mechanistic targets. The microbiota as a therapeutic target is reshaping the field of personalized medicine in this context. Identifying and investigating the function of novel microbial contributors to oncogenic processes has significant potential for the development and implementation of microbiome-targeted interventions, offering new avenues for prognosis and treatment.
